# Strengthening Exercises Improve Symptoms and Quality of Life but Do Not Change Autonomic Modulation in Fibromyalgia: A Randomized Clinical Trial

**DOI:** 10.1371/journal.pone.0090767

**Published:** 2014-03-20

**Authors:** Maria Bernadete Renoldi Oliveira Gavi, Dalton Valentin Vassalo, Fabian Tadeu Amaral, Danielle Constância Felício Macedo, Pablo Lúcio Gava, Eduardo Miranda Dantas, Valéria Valim

**Affiliations:** 1 Laboratory Assessment, Conditioning and Rehabilitation, Rheumatology Division, University Hospital of Federal University of Espirito Santo, Vitória-ES, Brazil; 2 Post Graduation Program in Physiological Sciences, Federal University of Espírito Santo, Vitória-ES, Brazil; 3 Laboratory Assessment, Conditioning and Rehabilitation, Physical Education and Federal University of Espirito Santo, Vitória-ES, Brazil; 4 Center of Health Sciences, Federal University of Espírito Santo, Vitória-ES, Brazil; 5 Center of Health Sciences, Federal University of Espírito Santo, Vitória-ES, Brazil; 6 Collegiate of Biological Sciences, Federal University of Vale do São Francisco, Petrolina-MG, Brazil; 7 Rheumatology Division, University Hospital, Medicine Department, Federal University of Espírito Santo, Vitória-ES, Brazil; University of Sevilla, Spain

## Abstract

**Objective:**

Autonomic dysfunction is an important mechanism that could explain many symptoms observed in fibromyalgia (FM). Exercise is an effective treatment, with benefits potentially mediated through changes in autonomic modulation. Strengthening is one of the less studied exercises in FM, and the acute and chronic effects of strengthening on the autonomic system remain unknown. The objective of this study was to assess the chronic effects of strengthening exercises (STRE) on autonomic modulation, pain perception and the quality of life (QOL) of FM patients.

**Methods:**

Eighty sedentary women with FM (ACR 1990) were randomly selected to participate in STRE or flexibility (FLEX) exercises in a blinded controlled trial. The intensity of STRE was set at 45% of the estimated load of 1 *Repetition Maximum* (RM) in 12 different exercises. Outcomes were Visual Analog Scale (VAS) for pain, Heart Rate Variability (HRV) analysis, treadmill test, the sit and reach test (Wells and Dillon’s Bench), maximal repetitions test and handgrip dynamometry; and quality of life by the Fibromyalgia Impact Questionnaire (FIQ), the Beck and Idate Trait-State Inventory (IDATE), a short-form health survey (SF-36).

**Results:**

The STRE group was more effective to strength gain for all muscles and pain control after 4 and 16 weeks (p<0.05). The FLEX group showed higher improvements in anxiety (p<0.05). Both groups showed improvements in the QOL, and there was no significant difference observed between the groups. There was no change in the HRV of the STRE and FLEX groups.

**Conclusions:**

Strengthening exercises show greater and more rapid improvements in pain and strength than flexibility exercises. Despite the benefits in fitness, pain, depression, anxiety and quality of life, no effect was observed on the autonomic modulation in both groups. This observation suggests that changes in autonomic modulation are not a target tobe clinically achieved in fibromyalgia.

**Trial Registration:**

ClinicalTrials.gov NCT02004405

## Introduction

Fibromyalgia (FM) is reported in 2–5% of the generalpopulation [1]. This syndrome, which impairs the quality of life (QOL) and increases financial expenses for the patient and society [2], is difficult to treat. The hallmarks of FM include widespread pain, fatigue, sleep dysfunction, muscular strength loss and cardiovascular fitness impairment [3].

FM patients show sympathetic hyperactivity and abnormal vagal balance. High sympathetic tonus increases the resting heart rate and reduces the heart rate variability (HRV) [4], [5]. Autonomic dysfunction might be an important mechanism associated with FM and could explain the major FM symptoms, such as fatigue, morning stiffness, sleep disorders, paresthesias, vestibular syndrome, palpitations, irritable bowel syndrome and Reynaud’s phenomenon [6]. Interestingly, a correlation between autonomic dysfunction and symptoms severity or quality of life has been previously described [7].

Undoubtedly, exercise is useful for the treatment of FM [2], [8], [9]. Most clinical trials have focused on aerobic fitness. Few studies have shown strengthening (STRE) as safe and effective for FM [8], [9]. However, until recently, the physiological mechanisms through which exercise induces clinical improvements remain unclear, and it is likely that better autonomic control might be involved. It has been shown that exercise improves autonomic modulation in healthy subjects and diabetic and heart disease patients [10]. Little is known about the acute and chronic physiological effects of STRE on autonomic modulation in FM [11]–[13]. Previous studies with small sample sizes have demonstrated that STRE improves total power and cardiac parasympathetic modulation in women with FM [11], [12].

Hence, we hypothesize that strengthening exercises are beneficial and might improve autonomic modulation. The objective of this study was to investigate the chronic effects of strengthening exercises on the heart rate variability and disease symptoms in FM patients.

## Methods

The protocol for this trial and supporting CONSORT checklist are available as supporting information; see Checklist S1 and Protocol S1.

This study was a randomized, evaluator-blinded, controlled parallel clinical trial comparing the STRE to flexibility exercises (FLEX) in women with FM from outpatient clinic of rheumatology of Federal University of Espírito Santo. The following inclusion and exclusion criteria were used.

### Inclusion Criteria

Women, between 18 and 65 years old, who met the criteria according to the American College of Rheumatology, 1990 [14].

### Exclusion Criteria

- Cardiovascular, respiratory, metabolic, and rheumatic diseases that could limit exercise;
- Diseases associated with autonomic dysfunction, such as arterial hypertension, diabetes and coronary insufficiency;
- The use of medication, such as beta blockers, calcium channel blockers, and any other anti-hypertensive, anticonvulsants, non-tricyclic antidepressants, and opioid analgesics, including tramadol, cyclobenzaprine >10 mg/day, andamitriptyline >25 mg/day, which could interfere with cardiovascular or autonomic responses;
- Exercise within the last 3 months;
- Inability to understand the questionnaires;
- Positive treadmill test for myocardial ischemia; and
- Receipt of the social security benefits.

Patients who were not taking medications during the first appointment were requested to use only paracetamol at a maximal dose of 2 g/day during the four months of the treatment. No other medications were allowed.

### Randomization

The patients were sequentially randomized to STRE or FLEX groups according to the order of inclusion in the study. The first included patient was allocated to intervention and the second to control group and so on. The physician responsible for initial evaluation and inclusion also randomized patients to groups and obtained the consent form. The assessor was blinded to the group membership and. Only the study coordinator and 2 professionals (physical educator and physical therapist) were responsible for the training knew the group membership information.

Primary outcomes were visual analogic scale for pain and Heart Rate Variability. Secondary endpoints were fitness outcomes (treadmill test, the sit and reach test, maximal repetitions test and handgrip dynamometry) and quality of life by the Fibromyalgia Impact Questionnaire (FIQ), the Beck and Idate Trait-State Inventory (IDATE), a short-form health survey (SF-36).

### Exercise Protocol

All the patients underwent clinical and cardiological evaluations using the treadmill test (TT), before starting the exercise program. The exercise prescription in both groups was consistent with the recommendations of the ACMS [15], with 45 min bouts at 2 times a week for 16 weeks.

The STRE group received supervised progressive training in the standing and sitting positions using weight machines. The intensity was moderate, with an overload of 45% of the estimated 1 RM, calculated based on maximal repetitions [15]. Eight major muscle groups were trained (*quadriceps femoris, hamstrings, biceps brachii, triceps brachii, pectoral, calf, deltoid, and latissimusdorsi*) in 12 different exercises, with 3 sets of 12 repetitions (*leg Press, leg extension, hip flexion, pectoral fly, triceps extension, shoulder flexion, leg curl, calf, pulldown, shoulder abduction, biceps flexion and shoulder extension*). The exercise program for the FLEX group included the major muscles [16].

### Evaluation Tools

#### The evaluation of fitness improvement

The participants underwent periodic muscle strength and flexibility evaluations every 30 days (before and 30, 60, 90 and 120 days after training) to adjust the training intensity and monitor the clinical evolution. Two expert professionals, a physiotherapist and a physical educator, both trained for the application of the analysis tools, performed the evaluations.

The participants were subjected to the treadmill test (Ergo PC 13, Micromed version 2.3), the maximal repetitions test [15], handgrip dynamometry [17], and the sit and reach test (Wells and Dillon’s Bench) [18].

The treadmill test was performed to assess the cardiovascular risk and measure indirect oxygen consumption (VO_2_). A treadmill (Centurion 200, Micromed) was employed using a previously described ramp protocol tested under similar conditions [16].

#### Pain perception, function, quality of life and mood evaluation

The pain perception was evaluated using the visual analog scale (VAS) at the beginning of the assessment and every 4 weeks thereafter. The QOL and mood were evaluated at the beginning of the assessment, and at 8 and 16 weeks thereafter. The trained examiners were blinded to the groups.

The evaluation of the symptoms was performed using the Fibromyalgia Impact Questionnaire [19], a short-form health survey (SF-36) [20], and the Beck and Idate Trait-State Inventory (IDATE) questionnaire [21].

#### Heart Rate Variability (HRV) analyses

HRV analyses were performed in the time and frequency domains, before and at the end (16 weeks) of the study. The electrodes were positioned in the distal region of the superior and inferior limbs. A continuous electrocardiographic recording of 10 minutes was performed with the participants in a supine body position in a quiet environment under a controlled temperature (22–24°C) using a digital electrocardiograph (Micromed, sample rate: 250 Hz) and specific software (Wincardio 4. 4a), which generated a beat-to-beat R-R interval series from the selected lead, with higher amplitude of the R wave (typically D2).

The HRV analyses were performed using software developed in Matlab. The series were automatically pre-processed for the removal of ectopic beats and artifacts. The analyses in the time domain included the proportion of R-R intervals that differed in more than 50 ms of the adjacent intervals (pNN50) and the square root of mean of the sum of the squares of difference among the adjacent intervals (RMSSD). The power spectral analysis was performed through autoregressive modeling, using the Yule-Walker method with a Levison-Durbin recursive algorithm. The model order was adjusted to 16 in all analyses, consistent with Dantas et al (2012) [22]. The oscillatory components present in the time series were classified based on very low (VLF: 0–0.04 Hz), low (LF: 0.04–0,15 Hz), and high (0.15–0.40 Hz) frequencies. The values for the spectral indices were presented as normalized units. Normalization was achieved by dividing the spectral power of each oscillatory component by the total power of the spectrum minus the VLF power component. The LF/HF ratio was achieved by dividing the LF components by the HF components in normalized units [22].

#### Statistical analyses and ethical procedures

Considering both alternative hypothesis (H0) and main hypothesis (H1), sample size calculation was performed taking in account comparisons between media of VAS for pain from two fibromyalgia populations with same variance and same number of observations. We have used MedCalc Program to determine sample size, considering type 1 error of 5%, type 2 error of 20%. The used standard deviation of VAS for pain was previously published (16). These led to at least 58 participants to be randomized to two groups.

The normality of the results was tested using the Shapiro-Wilk test. *Student’s “t”-test* for paired samples was used to perform intra-group comparisons at different times, when the data were normally distributed, and the nonparametric equivalent of Student’s t-test (Wilcoxon test) was used when the data showed an asymmetrical distribution. To compare the data between the STRE and FLEX groups, ANOVA for repeated measures was used, followed by *Bonferroni’s post-hoc test.* Bilateral tests were carried out adopting a 5% level of significance. The statistical software used for the analyses were SPSS version 20.0 and Biostat 5.0.

Other outcomes were fitness measure by treadmill test, the sit and reach test (Wells and Dillon’s Bench), handgrip dynamometry; and quality of life by the Fibromyalgia Impact Questionnaire (FIQ), the Beck and Idate Trait-State Inventory (IDATE), a short-form health survey (SF-36).

This project was approved through the Ethics Committee of the Health Science Center of the Federal University of Espírito Santo, protocol n° 119/10. Written informed consent was obtained from all patients.

This study was not registered before enrolment of participants started because it is not necessary according national law. The authors confirm that all ongoing and related for this intervention are registered.

## Results

**Figure 1:**
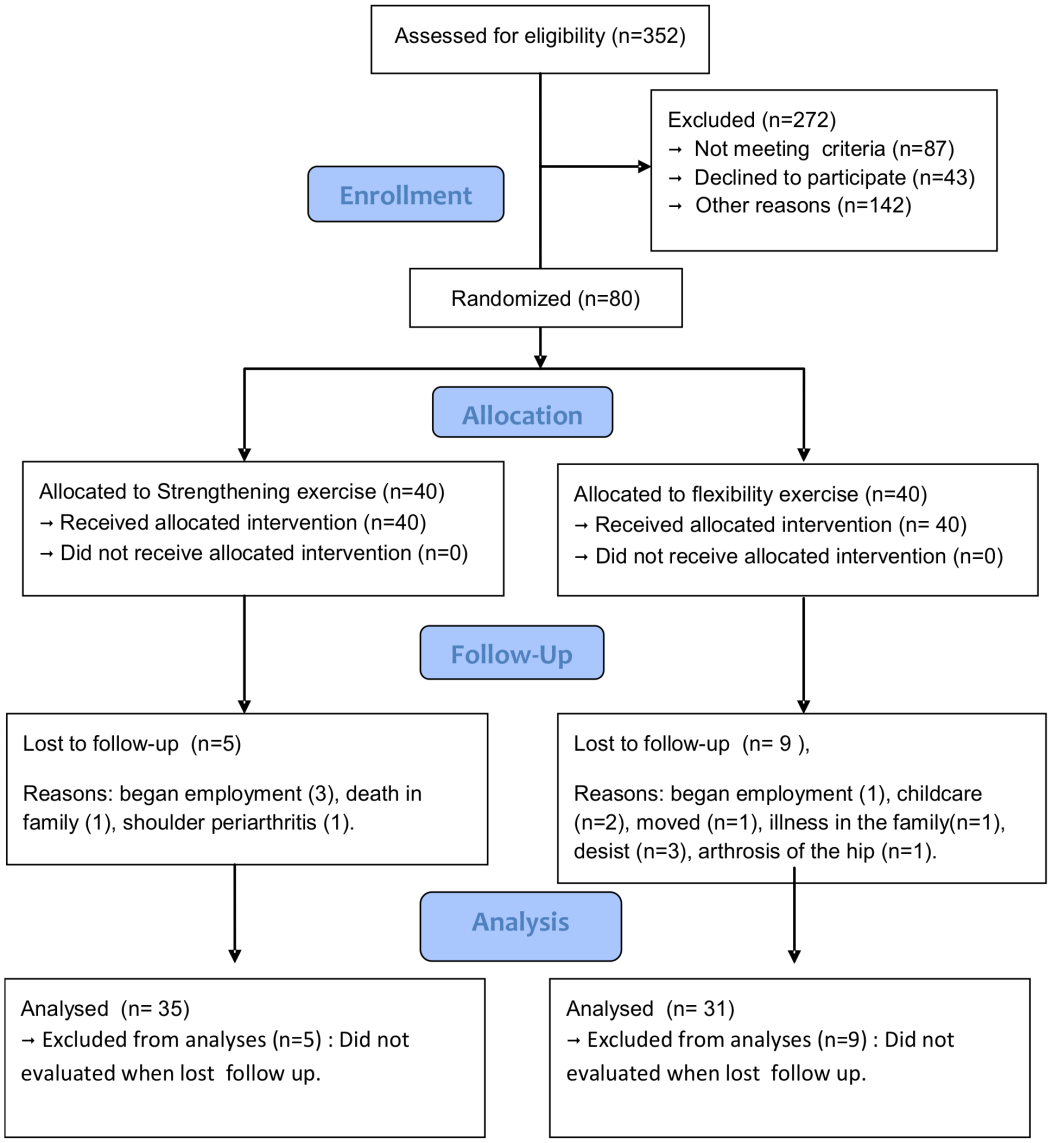
Study design and flow chart.

Eighty sedentary women between 18 and 65 years old, who met the criteria according to the American College of Rheumatology (1990) were included from July 2010 to September 2012. Training ended September 2012. Fourteen patients were excluded (9 patients in the FLEX and 5 patients in the STRE group) did not complete the study (Figure 1).

The mean of the analyzed sample (n = 66) had 46±8.5 years, and 56.6% of the participants were married, 62.8% were unemployed and 45.2% had 8 or more years of education. The more frequent symptoms included fatigue (97%), sleep disturbance (86.4%), paresthesias (80.3%), irritability (75.8%), cognitive dysfunction (74.2%), oral dryness (72.7%) and morning stiffness (71.2%).

The groups were similar in body weight, height, body mass index (BMI), maximal oxygen uptake (VO_2_), flexibility, pain perception, function (FIQ), life quality (SF-36), anxiety and depression. However, the STRE group was younger and stronger at the beginning of the study (Table 1). Approximately 7% of the patients (n = 9) in the STRE group and 8% (n = 8) of the patients in the FLEX group used low doses of cyclobenzaprine or amitriptyline.

**Table 1:** Demographics and clinical characteristics of treatment.

Variables	STRE (n = 35)	FLEX (n = 31)	p value
Age (years)	44.34±7.94	48.65±7.60	0.028*
Weight (kg)	65.75±9.30	67.70±13.63	0.504
Height (m)	1.59±0.07	1.56±0.06	0.061
CMI (kg/m^2^)	26.12±4.08	27.82±4.81	0.128
Pain (VAS) cm	7.81±1.59	8.38±1.46	0.184
FIQ	6.785	6.678	0.790
Depression (Beck)	25.83	22.78	0.225
Anxiety (IDATE TRAIT)	57.2	52.13	0.057
Anxiety (IDATE STATE)	46.69	44.45	0.407
SF-36 Physical Component	27.01	24.37	0.164
SF-36 Mental Component	33.47	36.98	0.181
VO2 max (ml/min)	31.20±10.87	28.90±9.82	0.372
Handgrip (kgf)	26.33±7.03	21.50±6.71	0.037*
Wells’ Bench (cm)	20.44±7.91	20.29±8.58	0.955

The values are presented as the means ± SD. STRE: strengthening exercise, FLEX: Flexibility exercise. FIQ: Fibromyalgia Impact Questionnaire. SF-36: Short-Form healthy Survey. VO2 max: maximal oxygen uptake. Wilcoxon Test: intra-group comparison. ANOVA: inter-group comparison, analysis of variance for repeated measures, p value: inter-group difference, *p≤0.05. *Pos hoc Bonferroni’s test*. Handgrip and Wells’ Bench data refer to N = 18 in the STRE group and N = 20 in the FLEX group.

**Figure 2:**
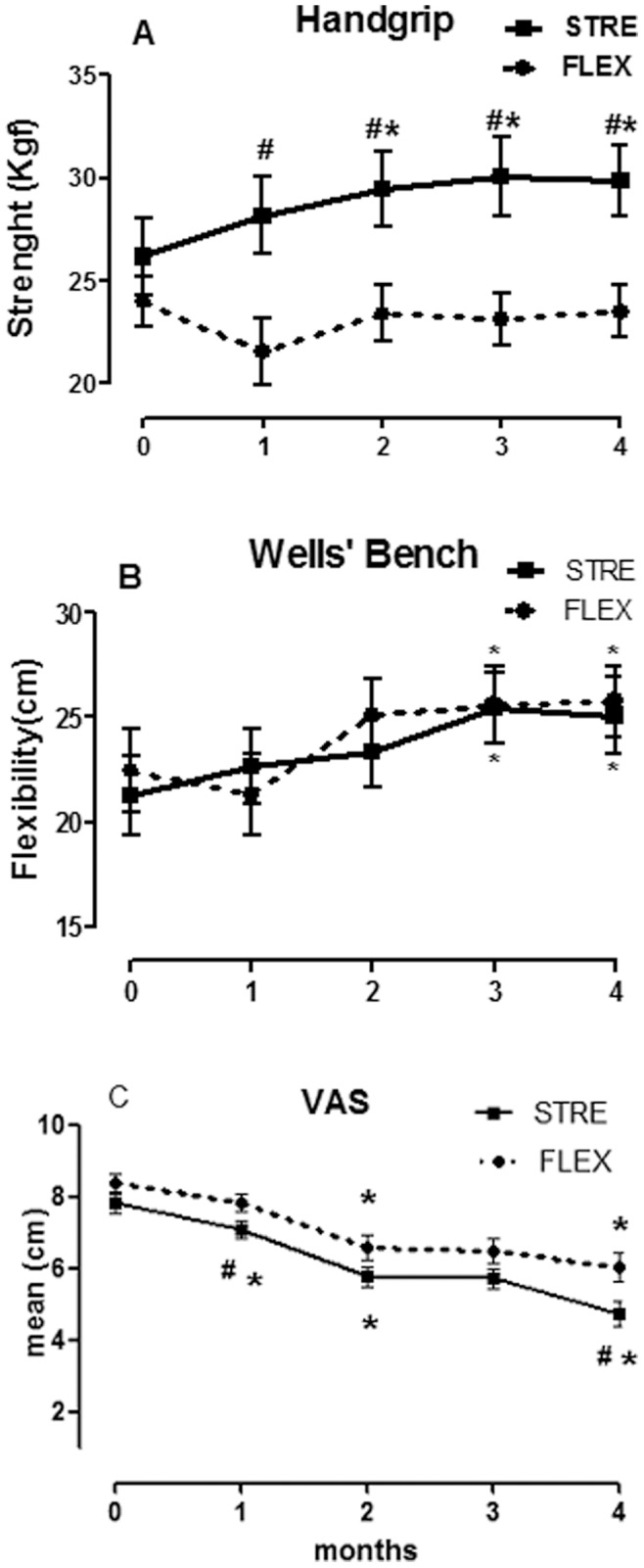
Isometric Strength, flexibility and pain evaluation. Mean and standard error of measures of strength (handgrip) (A), flexibility (Wells’ Bench) (B), Visual-Analogical Scale (VAS) (C), STRE: Resistance Exercise, FLEX: Flexibility Exercise. Wilcoxon Test: intra-group comparison (*), ANOVA: inter-group comparison (^#^), *p≤0.05. ANOVA: analysis of variance for repeated measures. Post-hoc Bonferroni’s test.

The strengthening and flexibility programs were effective, asimprove strength and flexibility were observed after treatment. The STRE group showed earlier and more gradual and progressive strength gain than the FLEX group for all muscle groups (results not shown) and in the global strength measured using handgrip dynamometry (Figure 2, Table 2).

**Table 2:** Physical fitness assessment at baseline and after 16 weeks.

	STRE before	STRE after	FLEX before	FLEX after	p value
	N = 35	N = 35	N = 31	N = 31	STRE × FLEX
SF 36 - Functional capacity	39±22.81	47.86±19.83*	29.39±16.64	43.39±19.85*	0.364
HF (beat/min)	90.97±11.87	88.49±12.30	90.13±16.54	87.87±11.20	0.833
VO2 max	31.20±10.87	37.71±6.24*	28.90±9.82	32.54±7.50	0.002*
Handgrip (kg)	26.33±7.03	29.75±7.04*	21.50±6.71	23.52±5.64	0.004*
Wells’ Bench	20.44±7.91	25.34±7.48*	20.29±8.58	25.80±7.56*	0.85
Shoulder flexion (kg)	7.29±2.00	11.09±3.08*	6.68±2.24	8.13±2.84	<0.001*
Leg (kg)	113.79±36.84	163.21±48.87*	100.43±35.96	132.24±50.55	0.013*

The values are represented as the means ± SD. STRE: strengthening exercise, FLEX: Flexibility exercise. Handgrip and Well’s Bench data refer to a subsample of 38 patients. Wilcoxon Test: intra-group comparison. ANOVA: inter-group comparison,

*p≤0.05. HF: heart frequency at rest. ANOVA: analysis of variance for repeated measures. p value: inter-group difference. *Pos hoc Bonferroni’s test*.

A progressive improvement in pain perception (monthly evaluation) was observed in the two groups, but the effect in the STRE group was higher than that the FLEX group after 30 days and at 4 months, indicating earlier improvement and better pain control in the STRE group (Figure 2).

The STRE group showed a significant improvement of fitness, not only in strength, but also in the maximal oxygen uptake. Neither of the groups showed any changes in the resting heart rate (Table 2). Both groups had good tolerance to exercise program and neither showed injury.

A significant improvement of the function, depression and QOL was observed in both groups. However, there was no difference between the groups, except for anxiety, in which the FLEX group showed better control (Table 3).

**Table 3:** Effects of exercise on function, symptoms, life quality, anxiety and depression.

	STRE before	STRE after	FLEX before	FLEX after	p value
	N = 35	N = 35	N = 31	N = 31	STRE × FLEX
FIQ	67.85±15.37	51.15±18.38*	66.78±17.24	51.15±18.38*	0.95
BECK	25.83±17.36	18.49±12.35*	22.77±18.56	16.39±9.46	0.452
IDATE-TRAIT	57.20±10.57	51.40±11.44*	52.13±10.67	45.19±11.74*	0.033*
IDATE-STATE	46.69±10.18	45.11±10.01	44.45±11.58	39.06±10.92*	0.022*
SF36- functional capacity	39.00±22.81	47.86±19.83*	29.39±16.64	38.39±19.85*	0.418
SF36- physical aspects	12.14±24.53	33.57±35.84*	9.68±26.36	28.23±38.59	0.414
SF36-Pain	27.68±13.49	42.68±14.82*	29.54±13.83	42.49±16.53*	0.994
SF36- General S. of Health	35.40±16.62	47.17±18.18*	45.58±20.43	56.65±20.93*	0.28
SF36-Vitality	25.14±17.38	41.14±20.15*	22.58±19.79	38.71±21.13*	0.533
SF36-Social aspects	41.76±21.40	55.66±17.92*	40.32±23.87	61.45±26.31*	0.306
SF36-Emotional aspects	27.62±40.81	40.00±41.07	35.48±41.22	51.61±43.75*	0.27
SF36-Mental Health	38.97±21.46	50.06±25.01	45.94±24.87	60.97±24.55	0.079
SF36- Physical Component	27.01±7.61	35.65±7.80*	24.37±7.58	34.15±9.20*	0.477
SF36- Mental Component	33.47±12.33	39.16±12.64	36.98±12.73	44.55±13.60	0.099

The values are represented as the means and ± SD. STRE: strengthening exercise, FLEX: flexibility exercise. Wilcoxon Test: p intra-group, ANOVA: inter-group comparison, analysis of variance for repeated measures,

*p≤0.05. p value: inter-group difference. Post Hoc Bonferroni’s test.

Although improvements in the symptoms were observed, no change in the autonomic modulation was detected after 16 weeks in both groups, as shown in table 4.

**Table 4:** Linear analysis of the heart rate variability before and after 16 weeks of training.

	STRE (n = 35)		FLEX (n = 31)		p value
	Before	16 weeks	Before	16 weeks	STRE x FLEX
Total power (ms^2^)	4095.81±1723.52	2884.52±1326.38	2414.78±785.46	1429.41±333.53	0.84
pNN50 (ms)	12.59±2.79	6.66±1.60*	5.99±1.52	5.51±1.46	0.75
RMSSD (ms)	48.40±10.31	39.16±8.62	36.86±7.28	30.37±4.50	0.96
LF (ms^2^)	1007.37±495.27	788.54±424.70	710.12±310.64	266.81±47.07	0.54
HF (ms^2^)	1680.60±797.25	1090.25±574.71	773.26±336.09	555.92±255.24	0.82
LFnu	42.80±3.03	43.42±3.53	43.68±3.72	46.7±4.48	0.73
Hfnu	47.24±2.92	47.83±3.40	43.85±3.83	44.83±4.16	0.73
LF/HF	1.36±0.23	1.88±0.56	1.88±0.51	2.73±1.00	0.64

The values are presented as the means ± SD. RMSSD: square root of the mean squared differences between adjacent normal RR intervals, in a time interval; PNN50: percentage of adjacent RR intervals differing longer than 50 ms; LF: low frequency; HF: analysis of variance for repeated measures,

*p≤0.05, p value: entre-group difference. *Pos Hoc Bonferroni’s test*. High-frequency, Wilcoxon Test: intra-group comparison, ANOVA: inter-group comparison.

## Discussion

The results showed that both treatments, strengthening and flexibility, improved the symptoms and QOL of patients with FM. However, in both groups, there was no interference in the autonomic modulation, as evaluated through heart rate variability.

Strengthening exercises were more effective and faster for pain control, whereas flexibility exercises were better for anxiety control. These results are useful to prescribe exercises for FM, as the effects of both treatments can be complementary. In addition, these differences can facilitate the selection of the most suitable exercises, according to the clinical profile of each patient. Only one previous study compared strengthening to flexibility, and the results showed that the magnitude of the effects was wider in the STRE group. Moreover, there was no difference between the groups for all variables studied [23]. Previous reports have compared strengthening with aerobic training [24]–[27], and similar results between the groups in the clinical improvement of the patients were observed. These studies are consistent with the results obtained in the present study, showing that STRE is beneficial, safe and comparable with other exercises [24]–[27].

In our study, the STRE group underwent training on machines and using free weights (barbells and dumbbells), and the load was adjusted monthly, similar to the regiment used for healthy sedentary subjects. Despite the low physical fitness, FM patientsexhibit muscle trainability and adaptation similar to healthy individuals, as previously reported [11]–[13], [28]–[32].

The STRE group was younger and stronger. But these factors did not affect the conclusion that STRE was more effective for strength gain and pain control, as demonstrated through intra-group and inter-group analyses. The STRE generated global fitness improvements, not only for strength but also for flexibility and oxygen consumption, likely reflecting the fact that strengthis essential in daily tasks.

Although effective for controlling symptoms, STRE did not affect the ANS, indicating that the involvement of other mechanisms, such as serotonin, endorphins, angiotensin II, cytokines, oxide nitric and GH increase and effects on the cerebral cortex [33]–[36]. The physiological effects varied according to the type of exercise. Thus, the production of serotonin, endorphins and increased autonomic modulation might be more influenced through aerobic fitness, as demonstrated in healthy and athletes [37]–[39]. It was recently demonstrated that serotonin is increased after 20 weeks of aerobic training, compared with FLEX training, in FM patients [35]. However, the mechanism underlying how exercise modifies the HRV remains unknown [10], [40], [41]. Until recently, studies have suggested that exercise influences HRV through neural stimulus by heart sympatho-vagal balance readjustments, with increased vagal modulation and reduced sympathetic modulation [42].

Few studies have focused on the acute and chronic physiological effects of strengtheningexercises on autonomic modulation in the FM [11]–[13]. It was demonstrated that after acute strengthening exercise, FM patients responded differently from the controls, as demonstrated by lower sympathetic and higher vagal modulation, likely reflecting altered autonomic responsiveness to physiological stress [12]. Only two previous studies concerning the chronic effects of STRE in FM, and the results are controversial [11], [13]. A previous study using a small sample (N = 10) demonstrated that STRE improves total power, cardiac parasympathetic tone, pain perception and muscle strength in women with FM after 16 weeks [11]. These same authors also reported that the HRV did not change after 12 weeks of strength training [13]. Other researchers have failed to demonstrate the interference of STRE in ANS in healthy people and athletes, consistent with the results of the present study, showing that the benefits of STRE are not associated with this physiological mechanism [41], [43], [44]. It is possible that, unlike aerobic training, STRE cannot modulate ANS. Beyond the type of exercise, another feasible explanation for this observation could be the high variability in the modulation of ANS in each subject [42]. Thus, a larger sample might be necessary to test this hypothesis, as high data dispersion could conceal the modulator effects of STRE training [41], [43], [44].

In conclusion, despite improvements in depression, anxiety and the quality of life in both groups, no effect of the strength training on autonomic modulation was observed, suggesting that autonomic modulation is not a target to achieve clinical benefits in fibromyalgia.

## Supporting Information

Checklist S1 CONSORT Checklist.
(DOC)

Protocol S1 Trial Protocol.
(PDF)
