# Supplementary material for: Strengthening Exercises Improve Symptoms and Quality of Life but Do Not Change Autonomic Modulation in Fibromyalgia: A Randomized Clinical Trial
Source: PLoS One. 2014 Mar 20;9(3):e90767. doi: 10.1371/journal.pone.0090767 (PMC3961245; doi:10.1371/journal.pone.0090767)
Supplement: Protocol S1 — Trial Protocol. (PDF) [file pone.0090767.s002.pdf]

FEDERAL UNIVERSITY OF ESPÍRITO SANTO

UNIVERSITY HOSPITAL CASSIANO ANTONIO DE MORAIS

RHEUMATOLOGY DIVISION

RESEARCH PROJECT – VERSION 1

TITLE:

THE STUDY OF HEART RATE VARIABILITY IN PATIENTS WITH FIBROMYALGIA - EFFECTS OF STRENGTHENING EXERCISES.

VITÓRIA

2010

SUMMARY

1 – INTRODUCTION..03

2 – OBJECTIVES...08

2.1 – MAIN...08

2.2 - SECONDARY..09

3 – CASUISTRY AND METHODOLOGY..09

3.1 – TYPE OF STUDY...09

3.2 – CASUISTRY..09

3.3 – EXCLUSION/INCLUSION CRITERIA...10

3.4 – STUDY PROTOCOL...11

3.5 – EXERCISE PROTOCOL……………………..12

3.6 – EVALUATION TOOLS..13

3.6.1 – GAIN EVALUATION OF PHYSICAL FITNESS..13

3.7 – PAIN, FUNCTION, QUALITY OF LIFE AND MOOD EVALUATION.............................15

3.8 – HEART RATE VARIABILITY (HRV) ANALYSIS……..16

4.0 – ETHICS..18

5.0 – FEASIBILITY CONDITIONS……………………………………………………………18

6.0 – CRONOGRAM…………………………………………………………………………19

7.0 – REFERENCES……………. in Portuguese version………………………………… 19

8.0 – APPENDIX....... ....   in Portuguese version..19

1 - INTRODUCTION

Fibromyalgia (FM) is a syndrome of widespread musculoskeletal pain which is characterized by the presence of at least 11 of 18 tender points, sleep disturbance and fatigue of chronic evolution (Wolfe et al., 1990; Brosseau et al., 2008). It is reported in 2-5% of the Brazilian population (Senna et al., 2005; Pereira et al., 2009) and represents 18.2% among the individuals with musculoskeletal non traumatic chronic pain in the state of Espirito Santo (Pereira et al., 2009). It affects 2 to 4% of the American population and the majority is women. People at any age may develop FM but they are more likely to have it between ages 35 to 50. (Heymann et al., 2006; Martinez et al., 2007). The prevalence of FM is probably greater than the one found in epidemiological studies. This is because these studies used the ACR 90 criteria which include the presence of tender points. (Wolfe et al.,1990). Nevertheless, there are many patients with fibromyalgia with diffuse pain without tender points and there are also patients with myofascial pain, making part of the fibromyalgia spectrum. In a Brazilian study the prevalence of FM defined by the diffuse pain with or without tender points was 5.5% (Pereira et al., 2009). In addition, this syndrome includes many symptoms which might fragment the evaluation and delay the diagnosis whenever some of them specifically prevails such as fatigue, pain, muscle weakness, memory problems, paresthesias, headaches, depression, anxiety, irritable bowel syndrome, dysmenorrhoea, subjective swelling, urethral syndrome, abdominal pain, sicca syndrome and they are all attributed to the autonomic nervous system dysfunction (Brosseau et al., 2008, Part 1; Hammond and Freeman, 2006; Gowans et at., 2002).

According to the majority of researchers (Penrod et al., 2004; Busch et al., 2007; 2009; Geel et al., 2002; Valim, 2003; Valim, 2006), the incapacity rate among people with FM is 46% and there are important social costs such as the direct ones (medicines, doctor’s appointments, lab and image exams) and the indirect ones (absences from work, job loss, the need of help to do the activities at home).

The eferent peripheral division of the Autonomic Nervous System (ANS) is represented by two components: sympathetic and parasympathetic which have antagonistic actions in the changes of body functions. This event enables the homeostasis maintenance particularly over the Heart Rate (HR) control.

In FM an abnormal sympatho-vagal balance is observed and Heart Rate Variability (HRV) is reduced. This fact increases the risk of cardiovascular morbidity and mortality (Coelho PF, 2008; Petzke and Clauw, 2000) .

Several clinical manifestations presented in FM such as fatigue, morning stiffness, sleep dysfunctions, anxiety, depression, sicca syndrome; irritable bowel syndrome and Raynaud's phenomenon could be explained by the dysautonomia. Due to these clinical characteristics which are compatible with ANS modulations, some studies associate the involvement of this system in the physio-pathogenesis of FM (Tak et al., 2009; Dogru et al., 2009; Coelho PF, 2008).

Several ways of measuring the autonomic activity have been studied and validated as:

1 – Electromyography of small fibers

2 – Cardiac Doppler under stress conditions and cold temperatures exposure

3 – Catecholamine dosages

4 – Orthostatic tests such as the Tilt Table Test

5 – Heart Rate Variability (HRV) through the evaluation system of the American European Task Force (Task Force, 1996)

6 – COMPASS questionnaire (The Composite Autonomic Symptom Scale)

The proposal of autonomic dysfunction in the main role in the pathogenesis of FM is based on in several research lines. The most consistent works have support in analysis of Heart Rate Variability (Solano et al., 2009 ; Tak et al., 2009 ; Figueroa et al., 2008; Furlan et al., 2005; Martinez,2001).

This kind of analysis showed HRV alterations compatible with sympathetic hyperactivity in many studies. The excessive sympathetic activity is followed by a minor response to different kinds of orthostatic stressors (Ozgocmen et al., 2006; Furlan et al., 2005 ; Petzke and Clauw, 2000).

Solano et al., in 2009, demonstrated that there is a correlation between the questionnaires that measure the impact of quality of life in FM (FIQ) and the questionnaire that assesses the autonomic dysfunction (COMPASS). The severity of FM is related to the multiple painless symptoms related to different expressions of dysfunction of ANS. Such correlation suggests that the autonomic dysfunction is an essential characteristic of FM.

Patients with FM have a high score in the psychosomatic evaluation of COMPASS. This likely reflects the presence of anxiety and depression, two psychosomatic components which might lead these patients to overestimate their symptoms.

The symptoms of chronic diffuse pain, allodynia and paresthesias in FM are attributed to persistent sympathetic activity. The chronic fatigue may be explained by the “celling effect” of the sympathetic hyperactivity in addition to “desensibilization” of adrenergic receptor.

Nowadays the most stressful type of life might contribute for a continuing sympathetic hyperactivity and individuals with a system of degradation of catecholamine genetically slow may have a greater risk of developing FM (Vargas-Alarcon et al., 2007).

The recent Brazilian Consensus for the treatment of fibromyalgia (Heymann et al., 2010), based on evidence, establishes that patients with FM must be treated with tricyclic antidepressants (amitriptlyne or cyclobenzaprine) or selective antidepressants, preferably “dual”, either associated or not to anticonvulsants. Analgesics may be taken if necessary. Physical exercises and cognitive-behavioral therapy compose complementary treatment and might benefit some patients. The strategy to the ideal treatment is a multidisciplinary approach with the combination of pharmacological and non-pharmacological treatment modalities (Koulil et al., 2006; Heymann et al., 2010).

Over the last 20 years some clinical trials have been published about exercises on the treatment of FM. In 2008 an extensive review was concluded by a great team of researchers from Ottawa Hospital Research Institute which resulted in the Ottawa Panel Evidence-Based Clinical Practice Guidelines for Aerobic Fitness Exercises in the Management of Fibromyalgia: Part 1(Brosseau et al., 2008) and the Ottawa Panel Evidence-Based Clinical Practice Guidelines for Strengthening Exercises in the Management of Fibromyalgia; Part 2 (Brosseau et al., 2008). Both studies (Part 1 and 2) had the objective of providing information from the treatment outcomes with strengthening and aerobic exercises in FM with evidence level for the prescription guidance of rheumatologists, physiatrists, physiotherapists, occupational therapists, kinesiologists and other professionals who might contribute for the syndrome treatment. The review evaluated aerobic fitness (Dadabhoy and Clauw, 2006) and combined or associated programs to medication treatment or other non-pharmacological interventions. (Isomeri et al., 1993; Cedraschi et al., 2004; Sencan et al., 2004). Stretching exercises were used as control intervention in few studies (Jones et al., 2002; Nativig et al., 1998, Pollock et al. ,1998). Few evaluated strength training (Jones et al., 2002; Kingsley et al., 2005; 2009; Figueroa et al., 2008), all of them with a small sample size.

Strenghtening exercise is defined as isometric, isokinetic or with concentric/eccentric resistance with the purpose of increasing the maximal strength produced by a specific muscle or muscle group. It may be performed by the help of machines, free weights, balls (Rooks et al., 2002; Tritschler, 2003; ACSM, 2010).

The strength evaluation is specific for muscle groups that are being tested. It is difficult to generalize on the global strength from the result for a muscle group. Some authors believe that the test of 1 RM of supine lift is the best isolated test to predict the global dynamic strength. (Tritschler, 2003; ACSM, 2010). If two tests are used to assess the dynamic strength, the supine lift must be used for the strength of the superior part of the body and the “leg press” for the inferior part (Tritschler, 2003; ACSM, 2010).

Muscle strength is basic for daily activities. The muscle strength training can improve functional capacity, bone mass, fatigue and pain (Hakkinen, 2001;2002; Valkeinen et al., 2004; 2005;2008; Brosseau et al., Part 2, 2008).

Aerobic fitness is the most studied type of exercise and with the highest evidence level for the treatment of fibromyalgia. (Busch et al., 2007; Brosseau et al., Part 1, 2008). There are evidences that supervised aerobic fitness can benefit patients with fibromyalgia, improving pain and quality of life. Stretching exercises also showed benefits in the control groups in pre-post analysis. In relation to strengthening exercises, Ottawa Panel demonstrated that there are emerging evidences that they are safe and can be beneficial. However, there is a necessity of more clinical trials of strengthening exercises with a sample size more suitable in order to assess the clinical effects of them in the treatment of FM. Yet, the physiological and physio-pathogenesis mechanisms that explain the benefit of exercise in the control of chronic pain remain controversial in the literature.

Exercises have a potential to decrease the inactivity and lack of fitness which are factors theoretically related to the pain threshold reduction. They can also improve

associated psychological factors. Strength or strenghtening exercise training can help patients to perform aerobic exercise more easily. (Jones et al., 2002; 2009). Moreover, it is observed that fatigue, more than pain, worsens the quality of life in fibromyalgia. These reasons could explain the reason why strength gain might promote a better quality of life (Valkeinen et al., 2008; Busch et al., 2007).

There are preliminary non control studies with a very small sample size (up to 10 subjects) showing that strengthening exercises improve strength, cardiac parasympathetic tone, HR variability, muscle resistance and increase the pain threshold as well (Figueroa et al., 2008; Kingsley et al., 2009;2010).

Although physical exercises are the most suggested and studied complementary therapy, further studies are still necessary to indicate a better prescription plan that assess the clinical effects of strength training and the mechanisms involved in the therapeutic outcomes.

Our hypothesis is that strength training exercises using prescription parameters well defined and according to ACSM can improve pain, associated symptoms and quality of life and that heart rate variability modulation, namely a better autonomic control, is one of the involved mechanisms in the pain control promoted by this type of exercise in fibromyalgia.

2 - OBJECTIVES

2.1 - Main

- To study Heart Rate Variability (HRV) in patients with FM who underwent strengthening exercise program comparing to control groups that underwent flexibility and stretching exercises.

2.2- Secondary

- to assess the strengthening training outcomes in the improvement of symptoms, quality of life and HRV in patients with FM, compared to control group that underwent stretching and flexibility exercises.

- to assess whether there is an association between strength gain and improvement of symptoms in FM.

- to study parameters for exercise prescription in FM.

- To assess the association between HRV and strength improvement and HRV and clinical improvement.

3 – PATIENTS AND METHODS

3.1-TYPE OF STUDY: randomized, evaluator-blinded, controlled clinical trial comparing group with strengthening exercises to control group receiving treatment with stretching and flexibility exercises. Both groups will make use of cyclobenzaprine (muscle relaxant pharmacologically classified as tricyclic antidepressants) as well as simple analgesic (acetaminofen, dipyrone). No medication will be introduced.

3.2 – PATIENTS - sixty women will be enrolled from the outpatient clinic of the Rheumatology Service of University Hospital of UFES between 18 to 65 years old, classified as FM according to the ACR90 criteria (Wolfe et al., 1990), randomized and allocated in control or intervention groups. The sample size was calculated considering the comparison between the averages in the two populations with FM. For this kind of analysis we assumed that the variances will be the same and that the number of observations will be the same in both samples. We will regard as a main parameter the visual analogical scale (VAS) for pain, expected delta of improvement at least of 30%, statistical significance of 5% and power of study of 80%. We will make use of the standard-deviation by EVA for pain previously known in the Brazilian population with fibromyalgia (Valim et

al., 2003). The minimum size calculated from the above parameters was of 29 subjects for each group or a total of 58 subjects. We intend to select a greater number of subjects in case of losses.

3.3 - EXCLUSION AND INCLUSION CRITERIA:

3.3.1 – Inclusion criteria:

- Classification criteria for Fibromyalgia (ACR 1990)

- Sedentary subjects (without regular physical activity in the last 3 months)

- Women between 18 to 65 years old

3.3.2 – Exclusion criteria:

- Cardiovascular and/or respiratory diseases that might limit physical activity (COPD, Pulmonary Fibrosis, asthma moderate to severe, respiratory insufficiency);

- Organ dysfunction (renal, hepatic, coronary, pulmonary insufficiency);

- Arterial hypertension and diabetes mellitus;

- Uncontrolled thyroid disease;

- Autoimmune rheumatic diseases and/or non-autoimmune with symptoms that might limit the movement or physical effort;

- The use of beta blockers, calcium channel blockers and any other anti-hypertensive, anticonvulsants; non tricycles antidepressants; opioid analgesic including tramadol.

- cyclobenzaprine >10 mg/day, and amytriptiline > 25 mg/day;

- Performing or have performed exercise within the last 3 months;

- Inability to understand the questionnaires;

- Positive treadmill test for myocardial ischemia;

- Receipt of the social security benefits.

3.4 – STUDY PROTOCOL

The experimental group (STRE) will receive strengthening training in “Station for lifting weight exercises” (Flex Mega 8, Flex Fitness Equipment Brand) using the recommendations of the American College of Sports Medicine (ACMS, 2010), twice a week, during 45 minutes for 16 weeks. The control group (FLEX) will receive stretching and flexibility exercise training according to the protocol of prescription and previously tested and described in appendix F (Valim et al., 2003), twice a week, during 45 minutes for 16 weeks. The minimum number of patients per group will be 30. The randomization will be by arriving order of the patients at the Clinic, having 2 groups with a distribution at random one by one. There will be an examiner for the selection and unblinded randomization and another for the application of tools that will be blind. Patients will undergo clinical and cardiological evaluation with an exercise stress test (Ergometric test) request in all of them according to ACMS as one more method of evaluation for inclusion and exclusion.

The evaluation will be performed by a blind examiner, an expert physiotherapist in rheumatology and trained for the application of the tools at the beginning, after 30, 60, 90, 120 days from the treatment onset.

The measure for prescription and muscle strength gain will be by the method of maximal repetitions (Lindh et al., 1994; Tritschler, 2003; Brosseau et al., 2008; ACSM, 2010). The evaluations will take place at the beginning, 30, 60, 90, 120 days (final evaluation). Evaluations will be performed by the same blind examiner for the examined group. The training team will be formed by a physiotherapist, physical educator, and

experienced doctors at strengthening training and flexibility and stretching exercises and they must follow the following protocol.

3.5 - PROTOCOL OF EXERCISES

The strength training will be performed according to ACSM and will have a mild to moderate intensity representing 45% of the estimated 1RM obtained from the maximal repetition method. Eight major muscle groups will be trained (quadriceps femoris, hamstrings, biceps brachii, triceps brachii, pectoral, calf, deltoid, and latissimus dorsi) in 12 different exercises, with 3 sets of 12 repetitions (leg Press, leg extension, hip flexion, pectoral fly, triceps extension, shoulder flexion, leg curl, calf, pulldown, shoulder abduction, biceps flexion and shoulder extension). Theses 12 different exercises will be distributed in a composed circuit system of 2 sets of exercises: Set A ((leg Press, leg extension, hip flexion, pectoral fly, triceps extension, shoulder flexion) and set B (leg curl, calf, pulldown, shoulder abduction, biceps flexion and shoulder extension). Each set will be applied once a week alternatively. The exercises will be performed twice a week during 16 weeks.

During the training process a load increase will be performed in order to seek progressive muscle strengthening improvement in a controlled way and according to patients' clinical evolution. Blood pressure and heart rate will be monitored as well.

Strength and strengthening training will be performed at the Evaluation, Physical Conditioning and Rehabilitation Laboratory (LACORE) at the University Hospital in in Federal University of Espirito Santo.

We have chosen the frequency of twice a week because it is a frequency (for this kind of study) validated in the literature (Figueroa et al., 2008, Kingsley et al., 2009; 2010).

3.6 – EVALUATION TOOLS

3.6.1 – GAIN EVALUATION OF PHYSICAL FITNESS

3.6.1.1 – **Strength evaluation**: performed by the method of maximal repetitions, obtaining the estimate of 1 RM. The method consists in establishing a load based on previous experience. The submaximal load individually suggested to the subject will allow the execution of up to 20 repetitions (Guedes and Guedes, 2006). The test will be repeated if the subject surpasses the pre-established target zone. Intervals of 1 minute approximately will be done between each attempt. We will not try more than 3 attempts. Tests will be performed alternating lower and upper limbs. The maximal repetition method allows the physical effort to be smaller using sub-maximal weights and also the possibility of these weights to be moved/lifted by a greater number of repetitions, whenever compared to maximal weights which can be moved/lifted only by one repetition. The estimate value of 1RM can be calculated through an equation obtained from the value of maximal load and by the number of repetitions. We have opted for the load test by repetition replacing test 1RM in order to reduce damage incidence and muscle uneasiness induced by the stress of physical effort. It is possible by maximal repetition test to estimate through mediation of math adjustments, the maximal load corresponding to 1 RM based on the maximal number of repetitions and the maximal load proposed for the test. This proximity is based on the straight linear relation between the close to 60-100% proportions of load observed in the test of 1RM and the number of repetitions observed in the load test by maximal repetitions. Models of statistic regression indicate that the load corresponding to the test of 1 RM has a tendency to increase with a mean of 2 to 2.5% in each repetition observed in the test of maximal repetition. Hence, regarding a decrease proportion of 2% as a reference in each further repetition, the correlation could be expressed by the mathematics relation:

$$1RM = \frac{\text{Submaximal load}}{100\% - (2\% \times \text{Repetitions})}$$

In which:

1RM: estimation of the equivalent load to 1RM, expressed in Kg
Submaximal load : submaximal load selected in the maximal repetition test expressed in kg,
100%: load equivalent rate to 1RM
2%: load decline rate equivalent 1RM to each repetition made in the maximal repetition test; and
Repetitions: number of observed repetitions in the maximal repetitions test .

(Tritschler, 2003; Fleck and Kramer, 2006; ACSM, 2010)

3.6.1.2 – **Dynamometry** - by manual hydraulic dynamometer to measure the strength of arms using handgrip every 30 days of training. Patients will perform the tests with the dominant limb 3 times, with 1 minute of rest between each measurement. Patients will be oriented to remain standing and keeping the arm along the body, the wrist and forearm in a pronated position comfortably holding the dynamometer with the forearm and measure scale turned to the examiner. The mean of the 3 measures will be registered. Dynamometry will used as a parameter of global strength evaluation since it presents a good correlation with maximal strength in people not trained (Fleck and Kramer, 2006).

3.6.1.3 – **Flexibility** – with Wells and Dilon's bench – it will be used to evaluate the flexibility with flexion of the trunk ahead the hips with both legs stretched. The tests will be performed every 30 days during the 4 months of training. We will record the longest distance achieved in the set of 3 movements (Wells and Dillon, 1952).

3.6.1.4 – **Treadmill test** – it will be performed before the beginning of the protocol of exercises and at the end to assess the cardiorespiratory fitness and measure indirect oxygen consumption (VO2). The ECG during the treadmill test will be performed with an analog-to digital converter of signals, Ergo PC 13 model in Micromed 2.3 version with a simultaneous acquisition of three derivations and record with speed of 25 mm/s and amplitude of 10 mm/mV. A belt Centurion 200 from Micromed will be used as ergometer and ramp protocol previously tested in this disease will be adopted (Valim et al., 2003).

3.7- PAIN, FUNCTION, QUALITY OF LIFE AND MOOD EVALUATIONS

Pain evaluation will take place at the beginning and every 30 days. The evaluations with questionnaires will happen at the beginning and after 2 and 4 months of treatment. The examiners will be trained and blind for the intervention.

3.7.1 – **Pain:** Visual Analogical Scale of Pain (VAS) is used. It is expected that the subject makes the equivalence between the intensity of the pain and a numerical classification having 0 for the classification of “No Pain” and 10 for the classification of “Maximal intensity imaginable pain” (Guimarães, 1998). The numerical classification indicated by the patient (on a ruler with number from 0 to 10) will be marked on the registration sheet.

3.7.2 **– Fibromyalgia impact Questionnaire (FIQ)**, validated by Burckhardt in 1991. It is the most specific questionnaire for fibromyalgia. It is compose by 10 items that measure the symptoms: function, general well-being, capacity for work, sleep disturbance, pain, fatigue, morning tiredness, morning stiffness, anxiety and depression. Each item and the total score varies from 0 to 10 and a higher value indicates a greater impact of the disease (appendix B).

3.7.3 **– Health Survey Short Form-36 Questionnaire of health (SF-36)**, validated by

Ciconelli (1999) is a generic tool to assess health and quality of life. It better evaluates the physical incapacity and for the work. It has 36 items grouped in 8 scales which are: physical aspects, functional capacity, body ache, general state of health, vitality, social aspects, emotional aspects and mental health. The domains can be grouped in 2 physical and mental components. It presents a final score 0 to 100 in which zero corresponds to the worst general state of health and 100 to the best state of health. (appendix C)

3.7.4 – **Beck Depression Inventory**, validated by Gorenstein and Andrade (1996), it consists of a questionnaire with 21 items of multiple choices. It is compose by several items related to depressive symptoms such as hopeless, irritability and cognitions like blame or feelings of being punished as well as physical symptoms such as fatigue, weight loss and decrease of libido. The higher the score is the higher is the level of depression (Gorenstein et al., 1996). We will use the following cut points recommended for patients with affective disorders by the “Center for Cognitive Therapy”: smaller than 10 = without depression or minimum depression; from 10 to 18 = mild to moderate depression; from 19 to 29 = moderate to severe depression; from 30 to 63 = severe depression (apprendix D).

3.7.5 – **State-Trait Anxiety Inventory** (STAI – validated by Andrade, 1996) Questionnaire of self-evaluation divided in two parts: 1 – evaluation of trait-anxiety (referring to aspects of personality) 2 – evaluation of state-trait anxiety (referring to systemic aspects of the context). Each one of these parts is composed by 20 statements. STATE means how the person feels “at the moment” and TRAIT how she “generally feels”. The score of each part varies from 20 to 80 points and might indicate a low degree of anxiety (0-30), medium degree of anxiety (31-49) and high degree of anxiety (greater or equal 50) – appendix E.

3.8 – ANALYSIS OF HEART RATE VARIABILITY (HRV)

It will be performed at rest before the beginning of treatment (zero time), with 24 and 48 hours (late acute) after the first effort test (denominated A1 and C1 times); with 24 and 48

hours after a strength test after 2 months of the beginning of treatment (A2 and C2) and also with 24 and 48 hours after the strength test, at the end of 4 months of training (A3 and C3). The effort will be test of strength evaluation described in 3.6.1.2 item.

Before the acquisition of data, patients will be told not to drink alcohol, caffeine and smoke cigarettes 24 hours earlier.

The electrodes will be positioned in the distal region of the upper and lower limbs. A continuous electrocardiographic recording of 10 minutes will be performed with the participants in a supine body position in a quiet environment under a controlled temperature (22-24°C) using a digital electrocardiograph (Micromed, sample rate: 250 Hz) and specific software (Wincardio 4. 4a), which generated a beat-to-beat R-R interval series from the selected lead, with higher amplitude of the R wave (typically D2).

3.8.1 – ESPECTRAL OR LINEAR ANALYSES

The HRV analyses will be performed in time and frequency domains using software developed in Matlab. The series will be automatically pre-processed for the removal of ectopic beats and artifacts. The R-R intervals that differ in more than 20 % of the mean of all intervals will be removed. The analysis of time domain will include the proportion of intervals R-R which might differ in more than 50 ms of the adjacent intervals (pNN50) and the square root of mean of the sum of the squares of difference among the adjacent intervals (RMSSD). The spectral analysis will be performed through auto regressive modeling, using the Yule-Walker method with a Levison-Durbin recursive algorithm. The model order will be adjusted to 16 in all analyses. The oscillatory components present in the time series will be classified based on very low (VLF: 0-0.04 Hz), low (LF: 0.04-0,15Hz), and high (0.15-0.40 Hz) frequencies. The values for the spectral indices will be presented as normalized units. Normalization consists in dividing the spectral power of each oscillatory component by the total power of the spectrum minus the VLF power

component. The LF/HF ratio will be achieved by dividing the LF components by the HF components (Task Force, 1996).

4.0 – ETHICS

All patients will read and sign the written informed consent. This project will be submitted by the Ethics Committee of the Health Science Center of the Federal University of Espírito Santo

5.0 – CONDITIONS OF FEASIBILITY/BUDGET:

This study will be developed by a researcher with a Master degree in Rheumatology and medical specialty of physical medicine and rehabilitation, doctor at UFES, for doctorate purpose in the Program of Post-Graduation in Physiology at UFES under the orientation of Professor Dalton Vassalo and co-orientation of Professor Valeria Valim, doctor at the Department of Medical Clinic of the Center for the Health Sciences, UFES.

Technical team for support: physiotherapists, physical educators, graduating students from the physiotherapy course, medicine, physical education, social service, medical residents, nursing.

The study will be performed at the Rheumatology Division and Evaluation Laboratory, Physical Conditioning and Rehabilitation of the University Hospital, UFES. There are evaluation conditions, follow up and patients exams on site.

The exercise tests will be performed at Lafex (Exercise Physiology Laboratory of UFES) and at the Cardiology Division of the University Hospital, UFES. The exams of Heart Rate Variability will be performed at the Laboratory of cardiovascular pathophysiology, Department of Physiological Science under the Professor Jose Geraldo Mill's authorization.

6.0 - CRONOGRAM

Activities	**02-06/2010**	**07-12/2010**	**01 a 12/2011**	**01 - 06/2012 a 09/2012**	**09/2012 – 03/2013**
Specialized Literature review liand submission to the ethical comitee. Ethical comitee's approval	X				
Clinical Evaluation and patients 's enrollment to the program		x	x		
Training prescription		x	X	X	
Execution of exercise program		x	X	X	
Re-evaluation of subjected patients		x	x	x	
Outcomes analyses					X
Report to Ethical comitee					X

7.0 – REFERERENCES: in Portuguese version

Versão 01

09/04/ 2010
